# Effect of Age and Morphology on Live Birth Rate After Cleavage Stage Embryo Transfer

**DOI:** 10.1007/s43032-020-00249-9

**Published:** 2020-07-09

**Authors:** Michael Awadalla, Nicole Vestal, Lynda McGinnis, Ali Ahmady

**Affiliations:** 1 Division of Reproductive Endocrinology and Infertility, Department of Obstetrics and Gynecology, Keck School of Medicine, University of Southern California, Los Angeles, CA USA; 2 Keck School of Medicine, University of Southern California, Los Angeles, CA USA

**Keywords:** In vitro fertilization, Cleavage stage, Embryo transfer, Live birth rate, Predictive model

## Abstract

Accurate knowledge of the live birth rate for cleavage stage embryos is essential to determine an appropriate number of embryos to transfer at once. Results from previous studies lack details needed for practical use. This is a mathematical analysis and model building study of day 3 cleavage stage embryo transfers. A total of 996 embryos were transferred in 274 fresh and 83 frozen embryo transfers. Embryo morphology was divided into 4 groups based on number of cells and fragmentation percentage. Each embryo transfer was modeled as an equation equating the sum of the live birth rates of the transferred embryos to the number of live births that resulted. The least squares solution to the system of embryo transfer equations was determined using linear algebra. This analysis was repeated for ages 35 to 42 years old at oocyte retrieval. The best fit live birth rates per embryo in the age group centered on 35 years old were 29%, 13%, 10%, and 9% for embryos in the 8-cell with ≤ 5% fragmentation, 8-cell with > 5% fragmentation, 9–12 cell, and 6–7 cell groups, respectively. Cleavage stage embryos with fewer than 6 cells on day 3 had very low best fit live birth rates close to 0% at age 39 years and were excluded from the primary analysis to prevent overfitting. These live birth rates can be used with a simple embryo transfer model to predict rates of single and multiple gestation prior to a planned cleavage stage embryo transfer.

## Introduction

It is challenging to determine the optimal number of cleavage stage embryos to transfer at one time. Current recommendations from the American Society for Reproductive Medicine suggest an upper limit of embryos to transfer at once based on age, embryo stage, and embryo prognosis. Individual clinics are encouraged to use their own data to aid in this decision in order to minimize multiple gestations [1]. Although there is limited information on how to develop a quantitative model for predicting transfer outcomes, determining the live birth rate per embryo is an essential starting point. Once live birth rates per embryo are known, models incorporating factors that affect all embryos (such as uterine receptivity) can be used to predict rates of singleton, twin, and higher order multiple gestations after transfer of more than one embryo.

Determination of the live birth rate for individual cleavage stage embryos based on morphology poses three unique challenges that limit research on this topic. First, since cleavage stage embryos are often transferred in multiples, it is difficult to determine how individual embryo characteristics impact the live birth rate. Previously published studies have dealt with this by stratifying the analysis by single or double embryo transfer and omitting transfers of three or more embryos. Analysis of double embryo transfers is sometimes limited to embryos of the same grade or the grade of the embryo with the more advanced stage is used in the model for both embryos [2, 3].

Second, analysis is complicated by the large number of data points for each cleavage stage embryo (oocyte age at retrieval, cell number, fragmentation, and cell symmetry). The Society for Assisted Reproductive Technology Clinic Outcome Reporting System (SART CORS) collects detailed information on embryo morphology and allows clinics to assign embryos an overall grade of good, fair, or poor [4]. Since clinics may assign overall embryo grades differently, modeling studies using the overall grade are limited by a lack of standardization across clinics. Assigning the overall embryo grade based on the live birth rate for an embryo with given morphological characteristics is preferred.

Third, accounting for age-related fertility decline is a challenge. Many analyses use fixed age groups of < 35 years, 35–37 years, 38–40 years, 41–42 years, and > 42 years [2, 3]. With fixed age groups, data for patients at the end of an age range is less accurate because it is influenced by data from the opposite end. Since most age-related fertility decline occurs between age 35 and 40, patients 37 years of age experience a sharp drop in predicted outcomes when they turn 38 years old when these age groups are used.

Logistic regression has often been used to model embryo transfers but has several limitations. First of all, only dichotomous outcomes (dependent variables) can be incorporated in the model. The dichotomous outcome most often used is live birth or no live birth. In most models, differentiation between outcomes of singleton, twins, and higher order multiple births cannot be done. Logistic regression also limits use of independent variables in the model. For example, one analysis omitted double embryo transfers that resulted in a single live birth because in these scenarios it cannot be known precisely which embryo resulted in the live birth. As a result, the embryo transfer information could not be incorporated into the model [5]. Another logistic regression model only took into account transfer of one or two embryos. In this model, when two embryos were transferred, the morphology of the embryo with the most advanced stage was used; the separate morphologies of each embryo were not incorporated into the model [3].

Although single blastocyst transfer is a good option for many patients, transfer of one or more cleavage stage embryos may be desirable for poor prognosis patients such as those with embryos that failed to make it to the blastocyst stage in prior cycles. Current literature shows an increased risk of multiple gestations when more than one cleavage stage embryo is transferred [6]. With double cleavage stage embryo transfer, the rate of multiple birth can be as high as 30–50% while the rate of multiple birth with single cleavage stage embryo transfer is less than 2% [7–11]. Accurate prediction of the live birth rate for a specific cleavage stage embryo may help determine when elective single cleavage stage embryo transfer may be appropriate [12].

In order to safely limit multiple gestations after cleavage stage embryo transfers, the live birth rate per cleavage stage embryo needs to be more precisely determined. The first objective of this study is to apply new techniques to determine the live birth rate per cleavage stage embryo based on morphology and age at oocyte retrieval. The second objective is to use these rates to assign overall embryo grades of good, fair, and poor.

## Materials and Methods

### Study Population

**Fig. 1:**
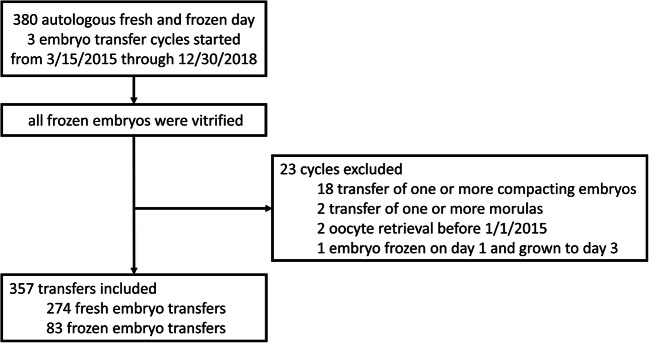
Flow diagram with inclusion and exclusion criteria

A model building analysis was performed using data from 357 fresh and frozen autologous cleavage stage embryo transfers in 267 unique patients at a single center. All day 3 embryo transfer cycles from March 15, 2015, through December 30, 2018, were included. Transfers were excluded if one or more compacting embryos or morulas were transferred, if oocyte retrieval occurred before January 2015, or if an embryo was thawed and grown to day 3 (Fig. 1). A total of 996 embryos were transferred for an average of 2.8 embryos transferred at once. The mean maternal age at oocyte retrieval was 38.9 (SD 3.8) years and the mean maternal BMI was 24.1 (4.4). Other maternal demographics and transfer cycle characteristics are shown in Table 1 and the embryo morphology distribution is shown in Table 2. The embryo transfers resulted in a total of 93 ongoing gestations (defined as a fetal heartbeat at 6 to 8 weeks of gestation) and 75 live born infants (61 singleton deliveries and 7 twin deliveries).

**Table 1:** Maternal demographics and transfer cycle characteristics

Maternal race/ethnicity	White	Asian	Hispanic	African American	Multiple	Unknown		
	44%	37%	11%	3%	4%	2%		
Transfer type	Fresh cleavage stage		Frozen cleavage stage					
	274/357 (77%)		83/357 (23%)					
Number of embryos transferred	1	2	3	4	5	6	7	8
Number of transfers (n = 357)	41 (11%)	126 (35%)	103 (29%)	52 (15%)	28 (8%)	4 (1%)	2 (1%)	1 (0.3%)

**Table 2:** Embryo morphology distribution of 996 embryos

Number of cells	2	3	4	5	6	7	8	9	10	11	12
Number of embryos	18 (2%)	22 (2%)	61 (6%)	76 (8%)	160 (16%)	136 (14%)	416 (42%)	33 (3%)	56 (6%)	2 (0.2%)	16 (2%)
Fragmentation	0%	1–4%	5%	10%	15%	20%	25%	30%			
Number of embryos	82 (8%)	119 (12%)	386 (39%)	215 (22%)	87 (9%)	71 (7%)	19 (2%)	17 (2%)			
Morphology grade	A	AB	B	BC	C	CD	D				
Number of embryos	2 (0.2%)	75 (8%)	690 (69%)	179 (18%)	49 (5%)	1 (0.1%)	0				
Morphology group	8 cells ≤ 5% fragmentation		8 cells > 5% fragmentation		9–12 cells		6–7 cells		2–5 cells		
Number of embryos	270 (27%)		146 (15%)		107 (11%)		296 (30%)		177 (18%)		

### Cleavage Stage Embryo Grading

At our center, day 3 embryos are assessed for number of cells, fragmentation, cell symmetry, and overall quality. Fragmentation is recorded as 0%, 1–4%, 5%, 10%, 15%, 20%, 25%, 30%, or > 30%. Morphology grade is assessed as follows: A for a symmetric blastomeres with proper cleavage rate (4 cells on day 2 and 8 cells on day 3) and no fragmentation; AB for equal size cells with <5% fragmentation or slightly irregular cells with no fragmentation; B for slow or fast cleavage, 5–15% fragmentation, or equal size with < 5% fragmentation and slightly irregular; BC for 16–30% fragmentation, extreme irregularity, or slow/fast cleavage rate with 5–15% fragmentation; C for > 30% fragmentation, extremely irregular cells with 5–15% fragmentation, or slow/fast cleavage rate with 16–30% fragmentation; CD for in between C and D; D for few blastomeric cells of any size and/or severe or complete fragmentation. A notation is made if embryos are partially compacting, compacting, or have reached the morula stage. Additional details on the in vitro fertilization protocols used are included in the Supplemental Methods.

### Determination of Live Birth Rates for Each Embryo Morphology Category

To determine the live birth rate per embryo, each of the 357 embryo transfers was modeled as an equation with unknown variables representing the live birth rate (LBR) per embryo for each category of embryo morphology. The coefficients (*N*) in the equation represent the number of embryos in each category that were transferred. The sum of the coefficients multiplied by their respective unknown LBR variables was set equal to the number of live births that resulted from the embryo transfer (Eq. 1). The equations were solved for the unknown variables with linear algebra to give the least squares solution to the system of equations using MATLAB version 9.5 (MathWorks). The MATLAB code used for the data analysis in this manuscript is being provided through Mendeley Data and can be accessed through the link in the reference [13].

1 $$ {N}_{\mathrm{category}\ 1}\times {\mathrm{LBR}}_{\mathrm{category}\ 1}+{N}_{\mathrm{category}\ 2}\times {\mathrm{LBR}}_{\mathrm{category}\ 2}+\dots =\mathrm{number}\ \mathrm{of}\ \mathrm{live}\ \mathrm{births} $$

### Moving Centered Age Groups

Live birth rates for each embryo morphology category were determined by age at oocyte retrieval in 1-year increments for patients aged 35 to 42 years old. For each age, we determined live birth rates based off of embryo transfers in patients that were 4 years younger to 4 years older giving us a 9-year age range centered on the age of interest. For example, the 32- to 40-year age range is centered on age 36 years. A 9-year age range was used because this was the smallest age range that smoothed out random variation in the data when analyzing 3 to 5 different embryo categories concurrently. Since the average age in each 9-year age group differed slightly from the original center age of interest, linear interpolation was used to re-center the live birth rates at the intended center age.

### Determination of Analysis Groups

The distribution of embryo morphological characteristics (Table 2) was reviewed to determine how best to group embryos for analysis of live birth rates. Morphology grade was determined to be less useful than number of cells and fragmentation since 69% of embryos were scored as a grade B. For this reason, morphology grade as assigned by the embryologist was not used in favor of the more quantitative measures of embryo cell number and fragmentation percentage. Embryos were first grouped by number of cells into categories of 2–5 cells, 6–7 cells, 8 cells, and 9–12 cells. This grouping put over 100 embryos into each category and showed a smooth decline in LBR with advancing oocyte age. Analysis with more categories based on cell number showed evidence of overfitting such as obvious signs of random error resulting in changes in LBR with age that were not biologically plausible.

There were 416 embryos with 8 cells and a decision was made to split this category up into two groups for ≤ 5% fragmentation and > 5% fragmentation. Other cell number categories were not able to be split up further because doing so gave results that did not fit the biological expectation that the LBR per embryo for a given category would smoothly decline with advancing age. An attempt was made to first group embryos by fragmentation percentage and then further by cell number but this produced results that were more difficult to interpret and appeared less biologically plausible.

**Fig. 2:**
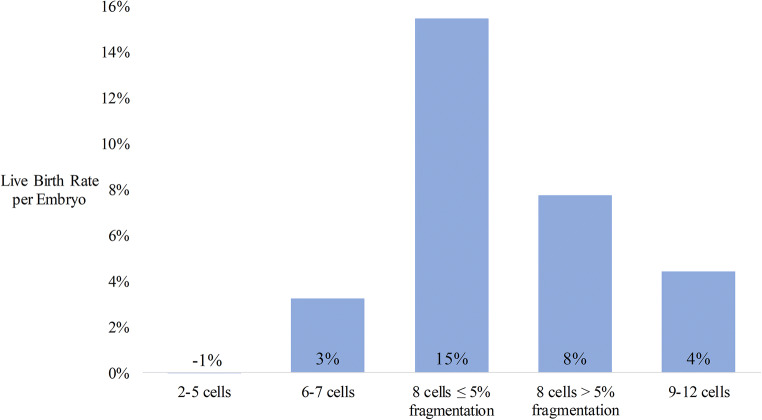
Best fit live birth rates per embryo by morphology group for all cleavage stage transfers

**Fig. 3:**
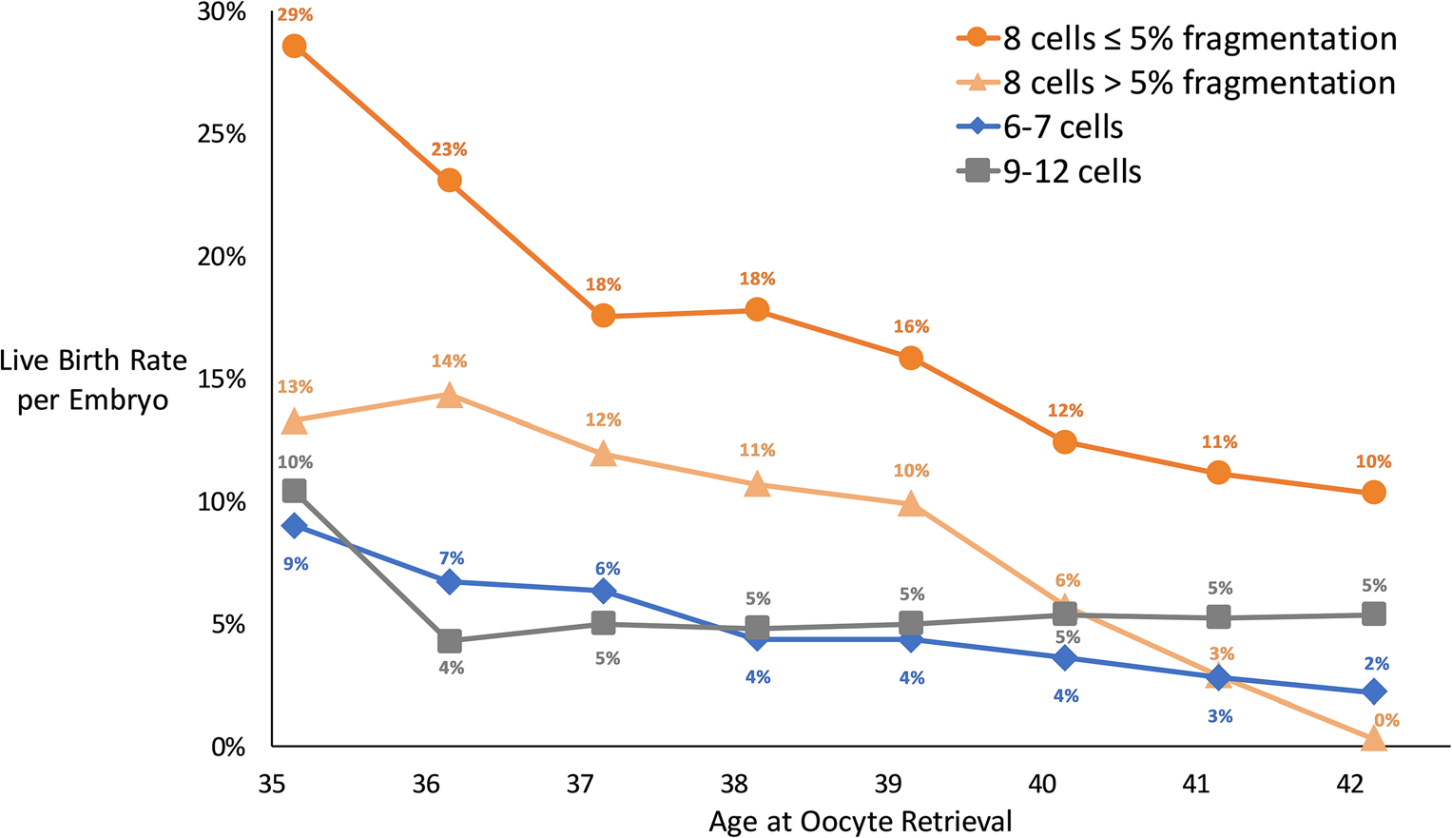
Best fit live birth rate per cleavage stage embryo by morphology group and age at oocyte retrieval. Nine-year moving age groups were utilized in the data analysis for this figure. Linear interpolation was used to center live birth rates on each integer of age

When analyzing all transfers for all ages together, the best fit LBR for the 2–5 cell group was − 1% which was interpreted to be likely close to 0 (Fig. 2). For this reason, the analysis with moving centered age groups was performed assuming that embryos with 2–5 cells do not significantly contribute to the LBR (Fig. 3). This step was determined to be important to prevent overfitting of the model.

### Statistical Analysis

Linear algebra was used to account for the number of embryos transferred and embryo morphology in the model. Stratification by age was achieved by using moving centered age groups to control for the confounding effect of age on the relationship between morphology and live birth rates. The results of the model are displayed graphically in Fig. 3 and were determined to fit the expectation of declining LBRs with increasing age for each embryo morphology group. The best fit results were consistent with those of other published studies.

## Results

The best fit live birth rates for all transfers (mean age 38.9 years) analyzed concurrently were 15%, 8%, 4%, 3%, and − 1% for embryos in the 8-cell with ≤ 5% fragmentation, 8-cell with > 5% fragmentation, 9–12 cell, 6–7 cell, and 2–5 cell groups, respectively (Fig. 2). The best fit live birth rates based on 9-year moving centered age groups are shown in Fig. 3. In the 35-year-old age group, the 8-cell embryos with ≤ 5% fragmentation had the highest best fit LBR (29%, good quality) followed by 8-cell embryos with > 5% fragmentation (13%, fair quality), 9–12 cell embryos (10%, poor quality), and 6–7 cell embryos (9%, poor quality). The 2–5 cell group was excluded from this analysis to prevent overfitting.

**Fig. 4:**
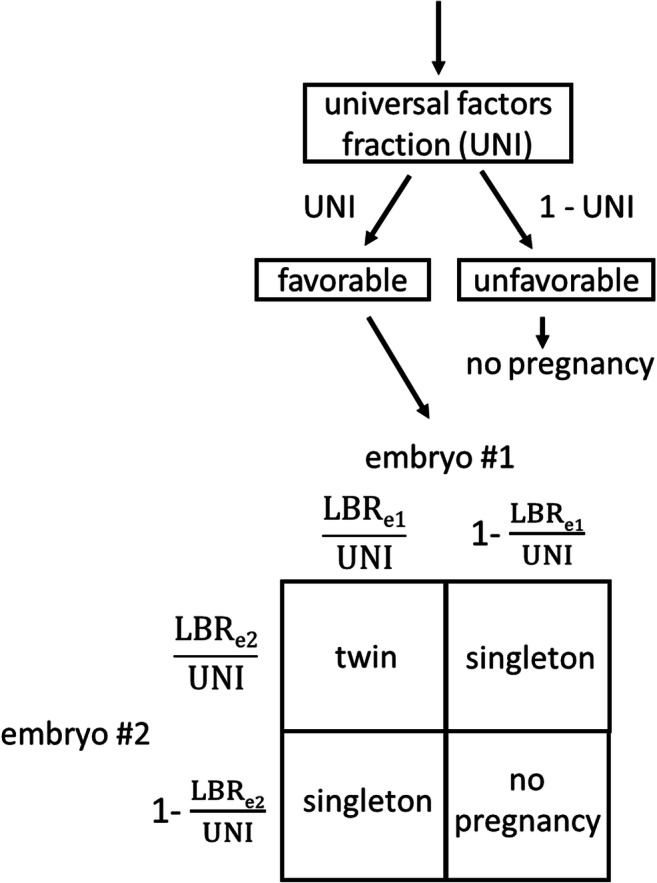
Logic for predicting outcomes of a double embryo transfer incorporating a universal factors fraction and live birth rates for embryo 1 and embryo 2. The universal factors fraction (UNI) is a fraction from 0 (never favorable) to 1 (always favorable). The probability of each outcome is equal to the product of the terms next to the corresponding arrows above and the corresponding terms on the perimeter of the square. For example, the probability of twins is UNI × $\frac{\mathrm{LBRe}1\ }{\mathrm{UNI}}\times \frac{\mathrm{LBRe}2\ }{\mathrm{UNI}}$. This same logic can be applied to transfer of more than two embryos. A UNI value of 0.70, representing adequate universal factors (such as uterine receptivity) 70% of the time, can be used as a best estimate for both fresh and frozen embryo transfers. UNI, universal factors fraction; LBR_e1_, live birth rate for embryo 1; LBR_e2_, live birth rate for embryo 2

We have previously validated an embryo transfer model based on the logic that if universal factors (such as uterine factors and others) are not favorable, no embryos will implant, but if universal factors are favorable, embryos are more likely to implant (Fig. 4). The model found favorable universal factors approximately 70% of the time which is consistent with the results of other studies [14, 15]. The model assigns LBR per embryo based on age at oocyte retrieval, cleavage or blastocyst stage transfer, and fresh or frozen embryo transfer. Due to the number of other factors considered, embryo morphology was not considered in the validated universal factors model. This current study is intended as an extension of that study to determine how cleavage stage embryo morphology affects the live birth rate per embryo.

In order to estimate the risk of multiples for a cleavage stage embryo transfer, the live birth rate per embryo and universal factors can be considered. For example, assume a 36-year-old has two day 3 embryos: one 8-cell embryo with 5% fragmentation and one 8-cell embryo with 10% fragmentation. From Fig. 3, the corresponding live birth rates per embryo are 23% and 14% respectively. The universal factors model suggests that 30% of the time universal factors are not favorable and neither embryo will result in a live birth. The remaining 70% of the time each embryo behaves independently of the other embryo with live birth rates of 0.23/0.7 and 0.14/0.7, or 33% and 20% respectively (Fig. 4). The end result is that incorporating universal factors does not change the live birth rate of an individual embryo but accounts for the increased risk of multiples when universal factors are favorable.

**Fig. 5:**
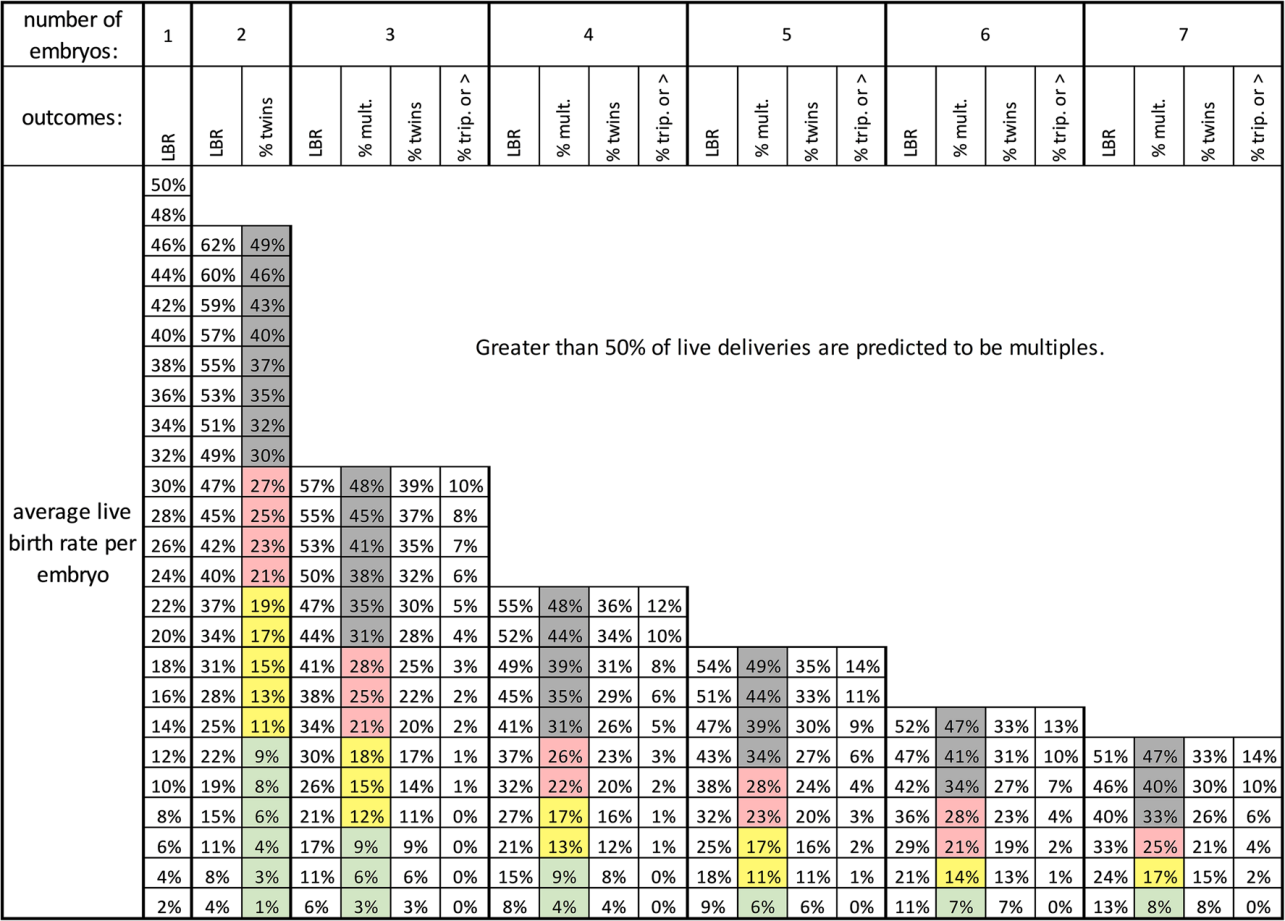
Predicted transfer outcomes by average live birth rate per embryo and number of embryos for a universal factors fraction of 0.70. The multiples column results are shaded green, yellow, red, or gray to indicate the risk of multiples at delivery (0–9%, 10–19%, 20–29%, or ≥ 30% respectively). LBR, total live birth rate per embryo transfer; % mult., percentage of live deliveries that are multiples; % twins, percentage of live deliveries that are twin deliveries; % trip. or >, percentage of live deliveries that are triplets or greater

To simplify calculation of the risk of twins, the average live birth rate of all transferred embryos can be considered. Using the average will make our calculation more conservative by slightly overestimating the risk of twins. For the above example, the average of 23% and 14% is 18.5%. A simple table based on the logic in Fig. 4 can be used to give the expected rates of live birth and multiple birth for any given combination of number of embryos transferred and average live birth rate per embryo (Fig. 5). For transfer of two embryos with an average live birth rate rounded to 18%, the table shows a 31% live birth rate with 15% of the live deliveries predicted to be twin deliveries. The exact predicted result without averaging the live birth rates or rounding is a 32% live birth rate with 14% of those deliveries predicted to be twins.

## Discussion

In order to assess the live birth rates from embryo transfers of single and multiple embryos, a system of equations approach is useful as it allows for solving with linear algebra. We grouped embryos into five groups for our analysis of live birth rate per embryo using all embryo transfers: 2–5 cells, 6–7 cells, 8 cells with ≤ 5% fragmentation, 8 cells with > 5% fragmentation, and 9–12 cells (Fig. 2). The best fit live birth rate for the 2–5 cell group of − 1% can have two interpretations. First, this may indicate that transferring these embryos decreased the live birth rate of other embryos. While this interpretation may be biologically plausible, we are not aware of any studies that support this possibility. The second possible interpretation is that the best fit live birth rate of embryos with 2–5 cells is close to zero. This is biologically plausible and is supported by other studies [3, 5]. With the linear algebra approach, an unknown variable with a true value of zero is never expected to have a best fit value of exactly zero. It is expected that the best fit value would be a small positive number 50% of the time and a small negative number 50% of the time. In the case here, a value of − 1% is interpreted to be a small negative number close to zero. The average age at oocyte retrieval in this study was 39 years old. It is likely that embryos with 4–5 cells on day 3 from younger patients have a small but clinically significant implantation potential. In order to prevent overfitting, embryos with 2–5 cells were omitted from our analysis based on age (Fig. 3).

Based on live birth rates per embryo, we determined how to best assign overall embryo grades of good, fair, and poor. Embryos consisting of 8 cells with ≤ 5% fragmentation (good) had the highest best fit live birth rates followed by 8 cells with > 5% fragmentation (fair) and 6–7 or 9–12 cells (poor) as shown in Fig. 3. Other studies have also found the highest live birth rates for embryos with normal cleavage rates (exactly 4 cells on day 2 or 8 cells on day 3) [5, 16]. Future database studies could consider using these same groupings for greater consistency across clinics rather than a subjectively assigned overall embryo grade. The SART CORS database records fragmentation in four categories: 0%, 1–10%, 11–25%, and > 25% [4]. 8-cell embryos with 0% or 1–10% fragmentation could be considered to have good overall quality and 8-cell embryos with 11–25% or > 25% fragmentation could be considered to have fair overall quality.

Using moving groups centered on the age of interest is more preferable than fixed age groups. Our analysis is repeated for a group of embryos centered at each age from 35 through 42. This allows us to derive clinically useful age-related information that can be used for patient counseling. We assume that errors from including a large age range are offset by centering the range on the age of interest. With this method, even large age ranges such as 9-year age groups introduce only small errors as long as the decrease in live birth rate over the age range is approximately linear.

Our study, as is the case in many studies of individual clinics, was limited by a small dataset. However, our methods were designed to optimize use of a small dataset through using a moving centered age group and including both single and multiple embryo transfers. To avoid overcomplicating the model, we only considered age, cell number, and fragmentation. Transfer cycle type (fresh or frozen cycle) was not able to be incorporated into the model because further stratification was not possible after accounting for age and four different morphological categories. Although our clinical data from a previous analysis shows a higher live birth rate per embryo with fresh cleavage stage embryo transfers compared with frozen cleavage stage embryo transfers, this is likely due to selection bias since the best embryos are selected for fresh transfers. Finally, it is unclear if using either aggregated data from multicenter databases or data from an individual clinic is applicable to other individual clinics. There may be limited ability to compare this data with that of other published studies because age may be reported or grouped differently between studies.

In conclusion, age and embryo morphology can be used to estimate the live birth rate and risk of multiples for cleavage stage embryo transfers. Incorporation of universal factors into the model is important because with multiple embryo transfers, there are factors affecting all embryos transferred together. These factors may result in higher rates of multiple gestation than if embryos implant independently. This model, which can be fit to data from other clinics, can help to more accurately guide clinicians and patients on the maximum number of embryos that can be safely transferred at one time. Since the applicability of data from one clinic to another is uncertain, if this data is used by other clinics, it should only be used to limit the planned number of embryos to transfer. We hope these methods and our results help the field of infertility move towards quantitatively assessing the risk of multiple gestations prior to every embryo transfer.

## Appendix

### Supplemental Methods

#### In Vitro Fertilization Protocols

##### Ovarian Stimulation

Ovarian stimulation was performed with recombinant human follicle-stimulating hormone (FSH) 75-300 IU SC daily (Follistim: Merck, Kenilworth, NJ; or Gonal-f: EMD Serono, Rockland, MD) and/or human menopausal gonadotropins (hMG) 75-150 IU SC daily (Menopur: Ferring Pharmaceuticals, Parsippany, NJ). Pituitary suppression was achieved through the use of gonadotropin-releasing hormone (GnRH) antagonist, GnRH agonist suppression, or GnRH flare suppression protocols. In antagonist cycles, the GnRH antagonist (0.25 mg SC ganirelix acetate daily or 0.25 mg SC cetrorelix acetate daily) was started when the lead follicle reached 14 mm in diameter at which time LH receptor stimulation was begun with SC hMG and/or 100 IU SC human chorionic gonadotropin (hCG) daily. When two lead follicles reached 18 mm in diameter or three follicles reached 17 mm in diameter, patients received a trigger medication for oocyte maturation and the GnRH antagonist was discontinued. 5000 IU hCG (or 10,000 IU hCG for patients with a weight of 200lbs or greater) was used for the trigger in GnRH agonist and GnRH flare protocols. With GnRH antagonist protocols hCG, leuprolide (4 mg SC every 12 h for 2 doses), or a hCG (2500 IU SC) and leuprolide co-trigger was used. Follicular aspiration was performed 34–36 h after the trigger administration. Doxycycline 100 mg PO twice a day was prescribed starting in the morning prior to the follicular aspiration and continued for 3 days for patients freezing embryos and until the morning of embryo transfer for patients receiving a fresh embryo transfer.

##### Oocyte Collection, IVF, and ICSI

Oocyte cumulus complexes were identified in the follicular fluid with an Olympus SZX10 microscope and transferred to oocyte collection dishes in Multipurpose Handling Medium with Gentamicin (MHM, Irvine Scientific, Santa Ana, CA). Oocytes were placed in MHM before insemination or ICSI. IVF insemination was carried out 4–6 h after the retrieval with each droplet being inseminated with 50,000 motile sperm. ICSI was performed on mature MII oocytes after removal of cumulus coronal cells. Removal of cumulus coronal cells was performed by exposure to hyaluronidase for 60 s followed by transfer to MHM and pipetting with 300-μm, 170-μm, 140-μm, and 130-μm diameter pipettes. ICSI was performed with an Olympus IX73 microscope microinjector (Olympus Corporation, Shinjuku City, Japan) with a heated stage (Tokai Hit, Shizuoka, Japan) and Narishige micromanipulator (Narishige, Tokyo, Japan). At 16–18 h postinsemination or ICSI, a fertilization check was performed.

##### Embryo Culture

Embryos were cultured in 15-μL drops of Continuous Single Culture Complete with Gentamicin and Human Serum Albumin (HSA) media (CSCM-C, Irvine Scientific, Santa Ana, CA) which was replaced with fresh media on day 4 of culture. Embryos were cultured in 38Special GPS Dishes (Life Global, CT, USA) overlaid with Liteoil (Life Global, CT, USA) for 3 days for cleavage stage embryos. The incubator conditions were set to temperature 37.0 °C, 5% O_2_, 8% CO_2_, and 87% N_2_. The % CO_2_ was titrated to maintain a culture media pH of 7.2–7.5 with a preferred pH range of 7.25–7.35. The embryos were cultured in either a Miri tabletop incubator (ESCO Medical, Changi, Singapore) or a HERAcell 150i incubator (Thermo Scientific, MA, USA).

##### Fresh Embryo Transfer

After follicular aspiration, patients were prescribed 2-mg estradiol PO twice a day until a positive serum HCG and 200-mg micronized vaginal progesterone twice daily until 10 weeks of gestation.

##### Embryo Vitrification

Cleavage stage embryos with 6–10 cells of grade BC or higher on day 3 were considered for vitrification. A Cryotip device (Irvine Scientific, Santa Ana, CA) was used for cleavage stage embryo vitrification.

##### Frozen Embryo Transfer

The majority of frozen embryo transfers occurred in programmed cycles. Starting on day 2 of the menses, a baseline transvaginal ultrasound was performed and 2-mg oral estradiol was administered twice a day for 6 days then three times a day until embryo transfer. On approximately day 11 of estradiol, a serum progesterone and endometrial thickness by transvaginal ultrasound were measured. Cycles were canceled if the progesterone was greater than or equal to 1.5 ng/mL. Additional days of estradiol were prescribed if the endometrial thickness was less than 7.0 mm. If the serum progesterone was less than 1.5 ng/mL and the endometrial thickness was equal to or greater than 7.0 mm, then IM and vaginal progesterone was started. IM progesterone was started with 50-mg progesterone in ethyl oleate starting at 9 p.m. (day 1 of progesterone). Subsequent days of progesterone consisted of IM progesterone at 9 a.m. and 200-mg micronized progesterone vaginally at 1 p.m. and 9 p.m. Cleavage stage transfers were scheduled on day 4 of progesterone (approximately 60 h after the first IM progesterone dose). Doxycycline 100 mg orally was prescribed twice a day starting in the morning the day prior to the embryo transfer for a total of three doses. After embryo transfer, patients were prescribed oral estradiol 2 mg twice a day, 50-mg IM progesterone in ethyl oleate once daily, and 200-mg micronized progesterone vaginally twice a day for 13 weeks. The first serum HCG was measured 11 days after cleavage stage transfers.
